# Modern Sociology

**Published:** 1902-12-27

**Authors:** 


### Modern Sociology.

### SCHOOLS AND SANITARY AUTHORITIES.

A large number of epidemics arise in schools. The majority of the schools where these epidemics arise are under the direct control of the State, for the number of children attending public elementary schools in England and Wales amounts to more than five and a half millions, or about one out of every six in the population. Why then does not the State do more to prevent and control these outbreaks? This was, in brief, the conundrum which was recently set before the Association of Medical Officers of Health by Dr. Meredith Richards, medical officer of health for Croydon, and also medical officer to the Croydon School Board. The dual position gives him a power, not possessed in equal degree by all his colleagues, to answer the question he put.

The importance of the question cannot be ignored, for young children are peculiarly subject to infectious diseases. Those who are attacked, even when they escape with their lives, are often left weak and unfit for the battle of life, and when, as often happens, the school is closed as a means to prevent the spread of infection, even those who escape the disease are sufferers by it, since it is the cause of their too short school life being shorn of some of its usefulness. But, unfortunately, there is no inevitable correlation between the sanitary and educational authorities, and though some favoured educational districts are under proper sanitary inspection, they enjoy this because their school boards are intelligent enough to see the importance of having a trained eye to look into the health of the children under their control. But a medical officer of health has no right, *ipso facto*, to enter a Board school and examine the children. Dr. Meredith Richards, indeed, seems to think that the teachers ought to be able to do part of the work. "Teachers should . . . be as familiar with the cardinal symptoms of infectious disease as they are with the multiplication table," he says. To this end he recommends that the teachers should be taught elementary hygiene. As a matter of fact, fully trained teachers have had some instruction in this subject, but, naturally, their teaching, which is to be passed on to their pupils, deals rather with the preservation of health than with the treatment of disease, and he would be indeed a believing soul who would expect that so much teaching as we may expect the teachers in elementary schools to receive and remember will make them "as familiar with the cardinal symptoms of infectious disease as with the multiplication table." Such teachers are, happily, to be found here and there, but we have no right to expect them universally. The business of teachers is education, not sanitation. But there are matters, directly educational, yet having a bearing on health, on which teachers should be better instructed than they are. They should pay more attention than is always done to the postures of the children under their care, should note and refer to the medical authority who should be attached to every school, those cases of marked stupidity which are too often due to mental deficiency, or else to faults of eye or ear, for which the child is not responsible. In most schools assistant teachers, at least, are allowed but little latitude in the matter of punishment; but even so, it can hardly be doubted that children are often punished for faults for which they cannot reasonably be blamed. This is within the teacher's province, because observation of such points requires time, and can be tested only at lessons; but there his mission ends. It is not for him to say what is the ailment from which a pupil is suffering. The results of such interference might occasionally be good, but would, for the most part, be the reverse.

What is really wanted is a control of schools by the sanitary authorities, which is not, as a rule, possessed by them. Lately, within the knowledge of the present writer, a sanitary inspector was sent to get from a teacher the list of absentees in her department, the reason of the inquiry being that a case of diphtheria had occurred in a child who had been attending the school. If all absentees were examined there was the possibility of other cases being discovered and dealt with, and, perhaps, a serious outbreak averted. But the teacher refused to give the names, and the officer had no power to insist on the information being given. If, in his irritation, the sanitary officer opined that the teacher hoped that the outbreak, if outbreak there was to be, would be of sufficient dimensions to justify the closing of the school, thereby securing for the staff a long holiday on full pay, the statement may be forgiven him. Without imputing to anyone a degree of selfishness which is little short of criminal, one may admit that teachers and school-managers often behave as if the sanitary authorities were their enemies instead of their best friends, and, in their anxiety to earn the highest possible grant, ignore all other considerations. If epidemics are to be nipped in the bud, as they might be in many cases, medical officers must have the right to go into a school and examine the pupils, and trace home the absentees whenever they think it necessary. This right is possessed by the medical officer of the borough of Eccles, thanks to the action of Dr. Crocker, who, in 1901, when medical officer of health there, managed to get a private Act passed giving him these powers.

On yet another point, more power should dwell with the medical authorities. That is the question of overcrowding. As things stand at present, if a school is certified as having cubic space for 500 scholars, 500 are permitted to attend it, without regard to their distribution. Now in schools, especially those in which education is carried on beyond the compulsory standards, the distribution of pupils is very unequal. The infant room may have 50 occupants, the other room where ex-VI. pupils are taught only ten. And while the one could hold more occupants, the other is overcrowded. The capacity of a school should be judged not as a whole, but room by room. Architects make some allowance for this difference in demand, but very often not sufficient, nor is there much hope of the matter being remedied until the sanitary authorities have much more say in the building of schools than is at present the case. It is not enough—though it is essential—that they should have control of the lavatory accommodation. That is not the only point of sanitary importance. The cleansing of many elementary schools is lamentably insufficient. The floors are swept daily, indeed, and therefore look passable; but sometimes they are washed only once a quarter, or when a convenient holiday arrives. It is doubtful if in every case the walls are washed down once a year. In country schools heating and ventilation are secured—not very well—by means of open fires and open windows. One is bound to make allowance for the defects of old buildings, but occasionally new schools are put up which perpetuate the faults of the old ones. This is, of course, more common in country districts, where the local authorities rather want to employ a local man than to go to the expense of employing an expert in school architecture. But there is no hope of improvement until the medical officer of the district has the right to examine and report on existing buildings and to see all plans for new ones. And if he is to do his duty well and fearlessly in this matter, he must be able to do it without either fear or hope of favour from local ratepayers. In short he must be independent of private practice. Does the Education Bill give us the hope that the powers of medical officers will be so extended, and made independent of local influence?

